# Magnesium Ion Acts as a Signal for Capsule Induction in *Cryptococcus neoformans*

**DOI:** 10.3389/fmicb.2016.00325

**Published:** 2016-03-15

**Authors:** Sudarshan S. Rathore, Thiagarajan Raman, Jayapradha Ramakrishnan

**Affiliations:** Centre for Research in Infectious Diseases, School of Chemical and Biotechnology, Shanmugha Arts, Science, Technology & Research Academy UniversityThanjavur, India

**Keywords:** biofilm, *Cryptococcus neoformans*, capsule, CAP gene, magnesium ions

## Abstract

Cryptococcal meningitis caused by *Cryptococcus neoformans*, is a common opportunistic neural infection in immunocompromised individuals. Cryptococcus meningitis is associated with fungal burden with larger capsule size in cerebrospinal fluid (CSF). To understand the role of CSF constituents in capsule enlargement, we have evaluated the effect of artificial CSF on capsule induction in comparison with various other capsule inducing media. Two different strains of *C. neoformans*, an environmental and a clinical isolates were used in the present study. While comparing the various capsule inducing media for the two different strains of *C. neoformans*, it was observed that the capsule growth was significantly increased when grown in artificial CSF at pH 5.5, temperature 34°C for ATCC *C. neoformans* and 37°C for Clinical *C. neoformans* and with an incubation period of 72 h. In addition, artificial CSF supports biofilm formation in *C. neoformans*. While investigating the individual components of artificial CSF, we found that Mg^2+^ ions influence the capsule growth in both environmental and clinical strains of *C. neoformans*. To confirm our results we studied the expression of four major CAP genes namely, CAP10, CAP59, CAP60, and CAP64 in various capsule inducing media and in different concentrations of Mg^2+^ and Ca^2+^. Our results on gene expression suggest that, Mg^2+^ does have an effect on CAP gene expression, which are important for capsule biosynthesis and virulence. Our findings on the role of Mg^2+^ ion as a signal for capsule induction will promote a way to elucidate the control mechanisms for capsule biosynthesis in *C. neoformans*.

## Introduction

Cryptococcal meningitis is a common, opportunistic neural infection in immunocompromised individuals (Satishchandra et al., 2007). It has emerged as a leading cause of morbidity and mortality in HIV positive and negative patients (Antinori, 2013). Recently, Center for Disease Control, USA, estimated the occurrence of approximately one million new cases of cryptococcal meningitis each year resulting in the death of 625,000 people worldwide (Park et al., 2009; CDC, 2015). The infection is acquired by inhalation of basidiospores, which resides in lungs and causes primary pulmonary infection, frequently asymptomatic. In immunocompromised host, the organism may disseminate either acutely or after a period of latency to extrapulmonary sites, with a particular predilection for the central nervous system (Bicanic and Harrison, 2004).

The etiologic agent, *Cryptococcus neoformans* has multiple virulence and predilection factors for invasion of central nervous system including capsule, melanin, phospholipase, mannitol, urease and proteinases (Buchanan and Murphy, 1998). The antiphagocytic polysaccharidic capsule is the prominent virulence factor of *C. neoformans*, which interferes with immunity and also serves as an antioxidant. The *C. neoformans* polysaccharide capsule structure are highly dynamic during budding or interaction with the host. The capsule undergoes changes in size and immunogenic properties as an adaptation in order to survive in the host (Vecchiarelli and Monari, 2012). GXM is the major component comprising 90% of capsule polysaccharide. *C. neoformans* shed substantial amount of exo-polysachharide during chronic infections resulting in profound consequences including resistance to host immune mechanisms, antimicrobial therapy and biofilm formation (Martinez and Casadevall, 2007). Recent studies reported the development of elevated intracranial pressure during excess release of exo-polysaccharide in CSF (Robertson et al., 2014).

During infection, the *C. neoformans* enlarges its size to form titan or giant cells. The increase in capsule size has profound consequences including, inhibition of phagocytosis, T-cell proliferation, and cytokine production. The giant cells have been reported in lung and CSF (García-Rodas et al., 2014). Cryptococcus meningitis is associated with fungal burden with larger capsule size in CSF which results in elevated intracranial pressure, less CSF inflammatory response and slower rate of fungal clearance (Robertson et al., 2014). The titan cells and capsule synthesis are regulated by cAMP dependent PKA. Significantly leading to PKA activation is also considered to be crucial for the generation of other virulence factors (Okagaki et al., 2011).

The activation of cAMP/PKA pathway increases the capsule biosynthesis and excess shedding of capsule favors the natural flocculation of *C. neoformans* cells to grow as a biofilm on various solid surfaces. The *C. neoformans* infections are becoming common due to the increased use of brain valve and other medical devices, such as ventriculoatrial shunt catheters, peritoneal dialysis fistula, cardiac valve, and prosthetic (Martinez and Fries, 2010; Liao et al., 2015). In fact, the increasing use of ventriculoperitoneal shunts to manage intracranial hypertension associated with cryptococcal meningoencephalitis highlights the importance of investigating the biofilm forming properties of this organism (Martinez and Casadevall, 2015). The biofilm formation is related to persistent infection as they resist the host defense mechanism and antifungal therapy. In addition to capsule, melanin production is another critical factor for pathogenicity, and melanized cells are more resistant to host cell response and antifungal therapy (Martinez and Casadevall, 2006).

Thus it is clear that increase in capsule size is vital for *C. neoformans* pathogenicity and virulence. Unfortunately it is difficult to achieve capsule growth under laboratory conditions as the mechanism for capsule enlargement remains unclear.

In the present study, analyses were performed for two different strains of *C. neoformans*, an environmental and a clinical isolate, to verify the responsiveness of the strains under different growth conditions for the expression of capsular polysaccharide.

## Materials and Methods

### Strains and Storage

Two *C. neoformans* strains were used in this study. The clinical *C. neoformans* was collected from Department of Microbiology, Government Medical College, Trichy, Tamil Nadu, India. The environmental strain was purchased from Microbial Culture Collection Centre at Chandigarh. (ATCC 14116). The cultures were maintained on potato dextrose agar slants and 20% glycerol stocks at –80°C.

### Capsule Induction: (Medium and Conditions)

To study the effect of various capsule inducing media, *C. neoformans* were cultured in PDB at 37°C for 24 h. Cells were washed with sterile PBS three times and resuspended in PBS. Cells were adjusted to 0.1 OD_600_ which is equivalent to ∼10^7^ CFU/ml and were inoculated in the following media: PDB, DMEM, MOPS [3-(*N*-morpholino)propanesulfonic acid], ACSF, ACSF+ 10% heat inactivated FCS. The media were maintained at different pH (5.5, 6.5, and 7.5) and temperatures (30, 34, 37°C). The capsule sizes were measured at three time intervals of 48, 72, and 96 h. Duplicates were maintained for the study (Frases et al., 2009b; Pettit et al., 2010; García-Rodas et al., 2014).

The preparation of ACSF media was as follows; [Stock I: 0.146 g NaCl (25 mM), 0.0186 g KCl (2.5 mM) in 10 ml double distilled water; Stock II: 0.015 g Na_2_HPO_4_ (1.25 mM) in 10 ml ddH_2_O; Stock III: 0.022 g CaCl_2_ (2 mM) in 10 ml ddH_2_O; Stock IV: 0.0120 g MgSO_4_ (1 mM) in 10 ml ddH_2_O; Stock V: 0.218 g NaHCO_3_ (26 mM) in 10 ml ddH_2_O]. All the 10 ml stocks were added in flask and made up to 100 ml with ddH_2_O to avoid precipitation, finally 0.1% glucose was added (Martinez and Casadevall, 2006).

The influence of individual components of ACSF [KCl (2.5 mM), CaCl_2_ (2 mM), MgSO_4_ (1 mM), and phosphate buffer (1.25 mM)] were tested for the induction of capsule growth in the presence of 0.1% glucose.

### India Ink Staining and Light Microscopy

A drop of India ink was mixed with an aliquot each of *C. neoformans* cultures on a glass slide. The samples were examined using Nikon (Eclipse C*i*-L) and images were taken with a camera (SLR, Canon D5100, Camera). To calculate relative size of capsule, diameters of whole cell, including capsule (Dwc) and cell body weight limited by cell wall (Dcb), were measured using ImageJ 1.48v software (National Institutes of Health, Washington, DC, USA). The size of the capsule relative to that of the whole cell was defined, as a percentage as {[(Dwc – Dcb)/Dwc] × 100}. Ten cells were measured for each determination and average was calculated (Frases et al., 2009a; García-Rodas et al., 2014).

### Biofilm Formation

*Cryptococcus neoformans* was grown in PDB for 24 h at 34°C (for ATCC *C. neoformans*) and 37°C (for clinical *C. neoformans*) in a rotary shaker at 150 rpm (to early stationary phase). The cells were collected by centrifugation and adjusted to 0.1 OD_600_ with each media (ACSF, ACSF+FCS, Ca^2+^ +Mg^2+^ +Glucose, Ca^2+^ +glucose, Mg^2+^ +glucose, DMEM, MOPS, and PDB). The cells were then inoculated on glass coverslip for 72 h at 34°C (ATCC *C. neoformans*) and 37°C (clinical *C. neoformans*). The biofilm was then washed thrice with PBS, to remove non-adherent cells and media from the cover slips and stained with India ink. The stained biofilms were then observed using Trinocular microscope (Nikon) at 40×. The experiments were done twice (Martinez and Casadevall, 2005).

### Capsule Gene Expression Analysis

#### Total RNA Extraction

For transcriptional pattern analysis, the ATCC *C. neoformans* and clinical *C. neoformans* were cultivated under following conditions, (i) PDB; (ii) PDB + Mg^2+^; (iii) MOPS; (iv) MOPS + Mg^2+^; (v) DMEM; (vi) DMEM + Mg^2+^; (vii) ACSF; (viii) Ca^2+^ + 0.1% glucose; (ix) Mg^2+^ + 0.1% glucose; (x) Mg^2+^+ Ca^2+^ + 0.1% glucose. Based on the dose dependent studies of Mg^2+^, 2 mM Mg^2+^, and 0.5 mM Mg^2+^ were selected for ATCC *C. neoformans* and clinical *C. neoformans*, respectively. After 48 h incubation time, *C. neoformans* were harvested and washed in chilled PBS. The RNA was extracted using RNAeasy mini kit (74104, Qiagen).

#### cDNA Synthesis

cDNA synthesis was performed in a total volume of 50 μl including 2–10 μg of RNA sample with one step RT-PCR Kit (210201, Qiagen). PCR amplifications were made from templates (1:20) with target primers (2:20), and (10:20) Taq DNA polymerase master mix (180301, Amplicon). Quantification of the transcript levels was performed using the ImageJ software, normalizing against β-actin.

#### PCR Analysis

The PCR reactions were performed using a Taq DNA polymerase 2.0X Master mix with 1.5 mM MgCl_2_ (180301, Amplicon). The protocols of the PCR were as follows: initial denature at 95°C for 10 min, followed by 40 cycles at 95°C for 30 s, annealing at 57°C (β-actin and CAP59), 55°C (CAP10), 60°C (CAP60), or 54°C (CAP64) for 30 s, and extension at 72°C for 30 s. Primer pairs for PCR are listed in **Table 1**. These primer pairs were confirmed to amplify the genome DNA fragments of both ATCC and clinical strains (Bolano et al., 2001). The relative concentration of each gene was analyzed by ImageJ software. All experiments were performed in duplicates.

**Table 1:** Primer used for CAP gene expression in ATCC and clinical *C. neoformans.*

Gene	Primer sequence (5′–3′)		Product size	Specification	Reference
CAP10	GGATGCAGAGTTGAGGAAGA	F	154	Glycosyltransferase	Okabayashi et al., 2005
	TATCCGTTGTAGGTCATGGC	R			
CAP59	CCGAACGAAGAAATCTCACT	F	244	Encodes to transmembrane protein	
	CTAGGTTGCATGTGTTCCCA	R			
CAP60	TCATGAGGGTGGAAACTACA	F	221	Unknown	
	GGGATGGATGAGTCTGAAAT	R			
CAP64	ATTGACTTTGATCGACGAGA	F	164	Gene regulation	
	ACTCTTCCTCGATCAATGTC	R			
β-actin	GCCCTTGCTCCTTCTTCTAT	F	156	Housekeeping gene	
	GACGATTGAGGGACCAGACT	R			
DNA ladder	Gene Ruler 1Kb DNA Ladder (SM0311)			250–10000 bp	

*F, forward primer; R, reverse primer.*

### Statistical Analysis

All experiments were performed in duplicates and the data analysis was done using Minitab 16 software. One-way ANOVA and Tukey’s test were performed to test statistical significance for multiple comparisons. All graphs were prepared with GraphPad Prism 6, and were expressed as mean ± experiments done in duplicates.

## Results

### Comparison of Capsule Induction in Various Medium

**FIGURE 1:**
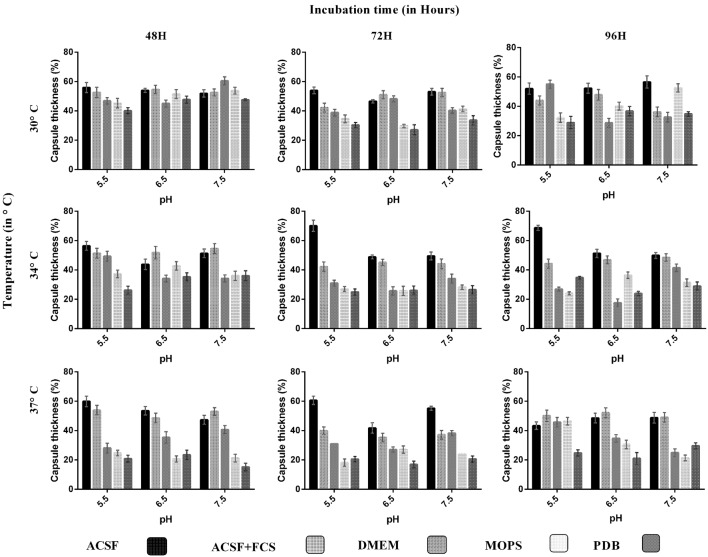
**Comparison of capsule induction in ATCC *C. neoformans* in different media (ACSF, ACSF+FCS, DMEM, MOPS, and PDB) at various pH, temperature, and incubation period.** The percentage of the capsule thickness [(total size^∗^ – cell size)/(total size^∗^) × 100] for ≥8 cells per group is shown. Bars represent standard errors (*p* < 0.0001). The greater capsule growth was observed in ACSF media (70%) at pH 5.5, 37°C, 72 h, followed by DMEM 60%, ACSF+FCS 55%, MOPS 53%, and PDB 47%. ^∗^Total size = capsule size + cell size.

**FIGURE 2:**
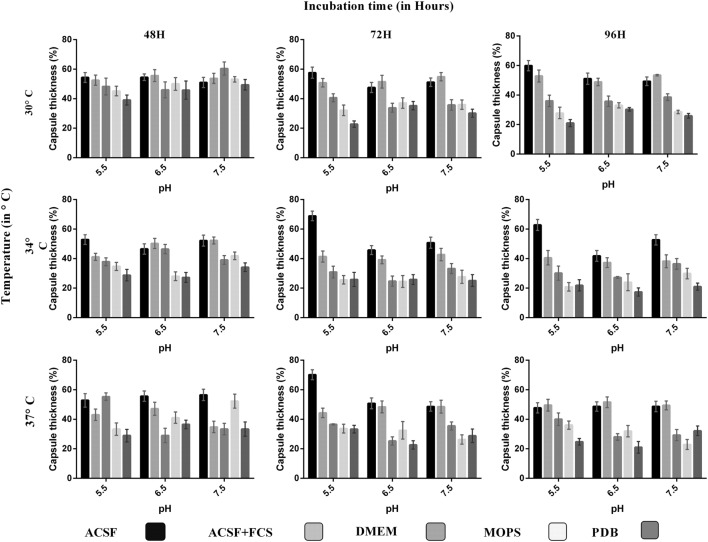
**Comparison of capsule growth in clinical *C. neoformans* in different media (ACSF, ACSF+FCS, DMEM, MOPS, and PDB) at various pH, temperature, and incubation period.** The higher capsule induction was observed in ACSF media at pH 5.5, 37°C, 72 h, which is followed by ACSF 70%, ACSF+FCS 56%, DMEM 55%, MOPS 53%, and PDB 49%. The cell size, capsule size, and percentage of the capsule thickness for ≥8 cells per group are shown. Bars represent standard errors (*p* < 0.0001).

We analyzed the effect of various nutrient based culture media for capsule growth and virulence trait expression for two different *C. neoformans* strains of clinical and environmental origin. While comparing the capsule growth in different media, it was found that ACSF significantly increased capsule growth in both ATCC *C. neoformans* and clinical *C. neoformans* compared to other media. The order of capsule growth for the strains are as follows: ACSF (69%) > ACSF+FCS (63%) > MOPS (62%) > DMEM (61%) > PDB (55%) and ACSF (68%) > ACSF+FCS (51%) > DMEM (49%) > MOPS (40%) > PDB (37%) for ATCC *C. neoformans* and Clinical *C. neoformans*, respectively. In both strains the capsule was induced by ∼32% when compared with the control media (**Figures 1** and **2**). Statistical analysis confirmed that ACSF was the best among the tested media (*p* < 0.0001; Supplementary Table 1).

The order of decrease in capsule growth in different medium shows that the capsule size decreases with increase in glucose concentration. Low glucose induces the production of cAMP and activates cAMP–PKA pathway (Pukkila-Worley and Alspaugh, 2004). However, single inputs are not sufficient for capsule induction, other inducing since signals are also needed (Zaragoza and Casadevall, 2004). Thus the effect of pH, temperature were also studied. All our test media contains considerable amount of salts and ions except PDB, which is observed as weakest medium for capsule induction. Serum induced capsule growth; however, the inducing effect was dependent on composition of the medium. Zaragoza and Casadevall (2004) observed capsule induction when grown in PBS with serum whereas not in sabouraud medium. Similarly in our study, 10% FCS induced capsule in the presence of ACSF. In summary, ACSF induced greater capsule growth in both the strains (Zaragoza and Casadevall, 2004).

### Effect of pH, Temperature, and Incubation Period

**FIGURE 3:**
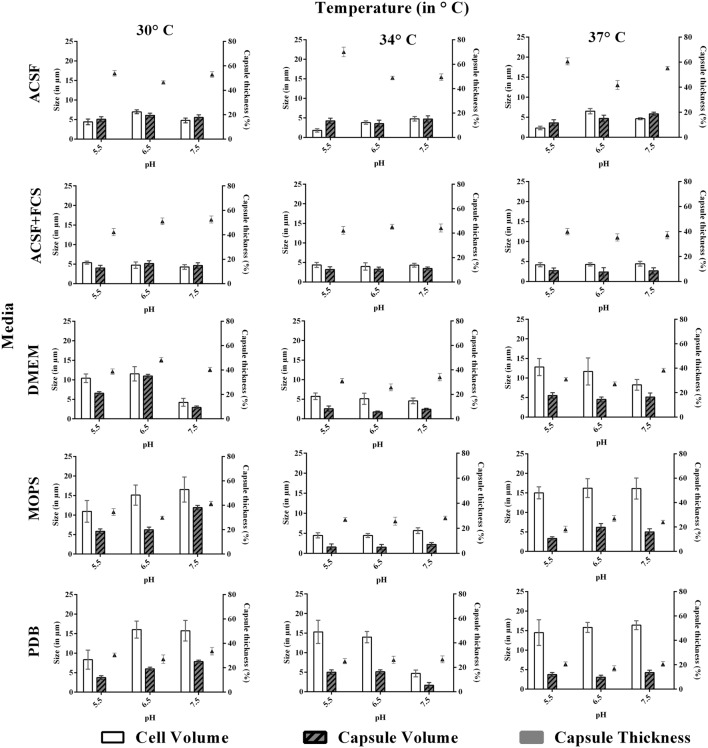
**Effect of pH and temperature on capsule induction in ATCC *C. neoformans* in different media at 72 h.** Left *Y*-axis represents size (in μm) for cell and capsule, compared with capsule thickness (in %) through right *Y*-axis. The cell size, capsule size and percentage of the capsule thickness for ≥8 cells per group are shown. Bars represent standard errors (*p* < 0.0001).

**FIGURE 4:**
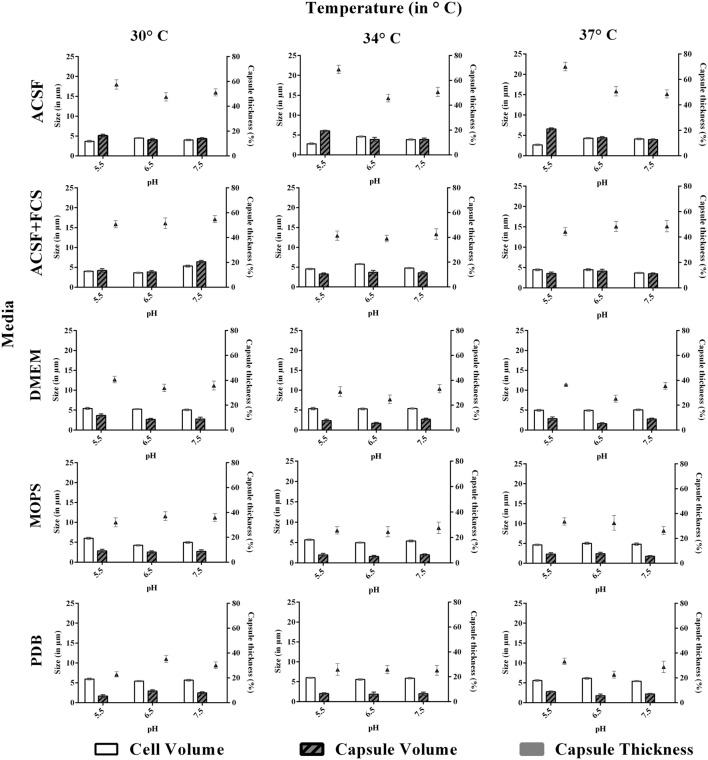
**Effect of pH and temperature on capsule induction in clinical *C. neoformans* in different media at 72 h.** Left *Y*-axis represents size (in μm) for cell and capsule, compared with capsule thickness (in %) through right *Y*-axis. The cell size, capsule size and percentage of the capsule thickness for ≥8 cells per group are shown. Bars represent standard errors (*p* < 0.0001).

We investigated the effect of pH, temperature and incubation period for the capsule induction in *C. neoformans*. To study the effect of pH, each media with different pH at 5.5, 6.5, or 7.5, based on PDB as control was selected for the study. From the results obtained, we observed a diverse range of capsule induction in different media. We achieved significant capsule induction (70%) for both the strains in ACSF with pH 5.5. A prominent increase in capsule size than the cell size for ATCC *C. neoformans* was noticed. Whereas, for other media, the capsule and cell size were relatively equal with capsule induction in the range of 34–54%. The ambient pH for capsule induction was observed to vary with the media types which are shown in **Figures 3** and **4**. As suggested by previous reports the effect of pH on capsule growth might be due to the alterations in cell wall stability, protein stability, morphogenesis, nutrient uptake (O’Meara et al., 2010) and changes in capsule structure (García-Rodas et al., 2011; Park et al., 2014). The exact mechanism by which pH regulates capsule growth is not clearly known. O’Meara and Alspaugh (2012) observed that there was no significant change in the expression of capsule biosynthesis genes at physiological pH. Effect of pH on the expression of capsule biosynthesis genes are required to elucidate the mechanism of capsule induction at different pH, which could be an important criteria for establishment and spread of infection (O’Meara and Alspaugh, 2012).

Similar to pH, the effect of temperature required for capsule induction was studied (**Figures 3** and **4**). From the results obtained it is clear that temperature does have an effect on capsule growth. However, we could not conclude whether an increase or decrease in temperature is influencing the capsule formation. ACSF induced capsule growth at 30, 34, 37°C but significant induction was at 34°C for ATCC *C. neoformans* and 37°C for clinical *C. neoformans*. However in our study, temperature has an effect on cell and capsule volume, postulating that the effect of physiological factors varies with type of strains used for the study.

We then investigated the effect of incubation period for capsule induction in *C. neoformans*. The study was conducted for a period of 96 h, and the capsule size was measured at regular intervals of 24 h. For both the strains, the optimum capsule induction period was 72 h when grown in ACSF at their optimum temperature (34 and 37°C) and pH (5.5). Thus it is clear that capsule induction appears to vary with respect to changes in media, temperature and pH. However, longer incubations did not significantly change the capsule size in both strains.

In summary, comparison of various capsule inducing media for the two different strains of *C. neoformans* shows that capsule growth was significantly increased when grown in ACSF at pH 5.5, temperature 34°C for ATCC *C. neoformans* and 37°C for Clinical *C. neoformans* and with an incubation period of 72 h. Using this information, we have made an attempt to understand the effect of individual components of ACSF on capsule induction.

### Evaluation of ACSF Components

**FIGURE 5:**
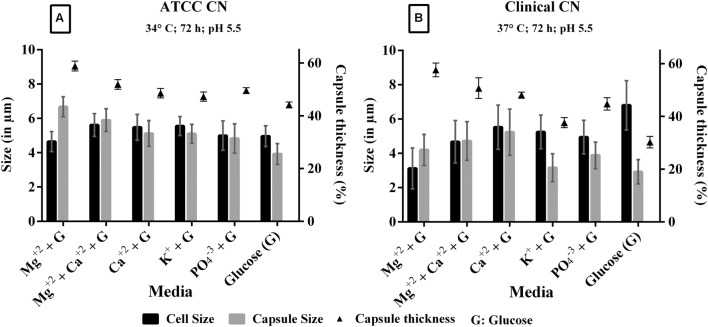
**Effect of ACSF salts [Magnesium (Mg^2+^), Calcium (Ca^2+^), Potassium (K^+^), Phosphate (PO_4_^3-^) salts were supplemented with 0.1% glucose] on capsule induction in **(A)** ATCC *C. neoformans* at 34°C, 72 h, pH 5.5; **(B)** clinical *C. neoformans* at 37°C, 72 h, pH 5.5. 0.1% glucose used as a control.** Left *Y*-axis represents size (in μm) for cell and capsule, compared with capsule thickness (%) through right *Y*-axis. The cell size, capsule size, and percentage of the capsule thickness for ≥8 cells per group are shown. Bars represent standard errors (*p* < 0.0001); G, Glucose.

The individual components of ACSF were analyzed to find out the most important capsule inducing factor. The defined medium has interesting components like KCl (2.5 mM), CaCl_2_ (2 mM), MgSO_4_ (1 mM), and phosphate buffer (1.25 mM). When each individual component of ACSF was supplemented with 0.1% glucose, we noticed Mg^2+^ as the prominent capsule inducing factor in both strains. The order of capsule induction for different salts for clinical *C. neoformans* is Mg^2+^ (58%) > Mg^2+^+ Ca^2+^ (52%) > Ca^2+^ (48%) > PO_4_^3-^(46%) > glucose (46%). For ATCC *C. neoformans*, Mg^2+^ (59%) > Mg^2+^+ Ca^2+^ (57%) > Ca^2+^ (54%) > PO_4_^3-^(52%) > glucose (50%) and no capsule induction was observed in KCl for both the strains (**Figure 5**). So we increased the pH of the KCl containing medium for capsule synthesis. Surprisingly capsule induction was observed at higher pH (pH 7.5). The effect of ACSF ions on capsule formation were studied by comparing the cell size and capsule thickness. From the results we obtained, it is very clear that *C. neoformans* when grown in the medium containing Mg^2+^ ion, show increased capsule production even though the cell size was relatively small. Whereas in case of effect of ions like K^+^, PO_4_^3-^, the cell size was greater than capsule. In the presence of Ca^2+^, the cell and capsule thickness were equal, whereas a significant induction was observed when Ca^2+^ medium was supplemented with Mg^2+^. The effect of Mg^2+^ ion in capsule induction is clearly evident in **Figure 5**. A similar kind of results was observed with clinical *C. neoformans*. However, in both the strains, Mg^2+^ significantly induced to capsule growth under any of the conditions tested, like different pH (pH 5.5, 6.5, and 7.5), temperature (30, 34, and 37°C), induction period (48, 72, 96 h). Statistically Mg^2+^ was found to enhance capsule compared with other salts. Through One-way ANOVA analysis Mg^2+^ was found to be the best salt compared to other ACSF salts (*p* < 0.0001; Supplementary Table 2). These results show the importance of Mg^2+^ in *C. neoformans* capsule growth.

There are earlier studies on *C. neoformans* capsule growth with some salts like NaCl, MgCl_2_, CaCl_2_, phosphate buffer, MOPS, FeS0_4_, (Lian et al., 2005; Nimrichter et al., 2007; Robertson et al., 2012; Kretschmer et al., 2014). High concentrations of NaCl inhibited the capsule growth suggesting that Na neutralizes the negatively charged GlcA residues (Nimrichter et al., 2007). A recent study by Kretschmer et al. (2014) reported that phosphate ions are helpful for the nutrition acquisition and influence capsule synthesis. The author observed that phosphate uptake is critical for capsule synthesis as it is integrated with the key signaling pathways for nutrient sensing. The effect of iron on capsule induction is varied with the induction being greater in both iron limited and iron rich condition, like brain tissue. The role of iron, however, in capsule induction is not clearly understood (Kretschmer et al., 2014).

The three major events in *C. neoformans* capsule formation includes, capsule synthesis, assembly and degradation (Bose et al., 2003). Once the capsule components are synthesized they are assembled to form a thick outer covering to form mature *C. neoformans*, which requires the involvement of multiple regulatory pathways. The dynamics of capsule size is affected by the varied environmental cues. There are reports suggesting the role of divalent ions (Mg^2+^, Ca^2+^) in capsule assembly and capsule growth (Nimrichter et al., 2007; Robertson et al., 2012) and they are dose dependent. Increasing the concentration of divalent ions decreases the capsule assembly. In our study the effect of Mg^2+^ alone is significantly greater than Mg^2+^ + Ca^2+^, suggesting its vital role in capsule synthesis.

### Magnesium Acts as Signal for Increased Capsule Production and Dose-Dependency

**FIGURE 6:**
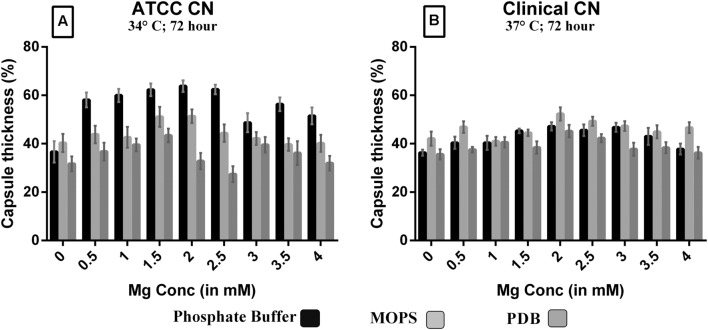
**Effect of Mg^2+^ with phosphate buffer, MOPS and PDB on capsule induction with increasing Mg^2+^ concentration (0.0 – 4.0 mM) in ATCC *C. neoformans* at 34°C, 72 h, pH 5.5 **(A)**; clinical *C. neoformans* at 37°C, 72 h, pH 5.5 **(B)**; 0.1% glucose used as a control.** *Y*-axis represents capsule thickness (in %). The higher capsule size was observed with phosphate buffer + 2.0 mM Mg^2+^ in ATCC *C. neoformans* (67%) whereas in clinical *C. neoformans* (58%) sample, PDB + 0.5 mM Mg^2+^. The cell size, capsule size and percentage of the capsule thickness for ≥8 cells per group is shown. Bars represent standard errors (*p* < 0.0001).

**FIGURE 7:**
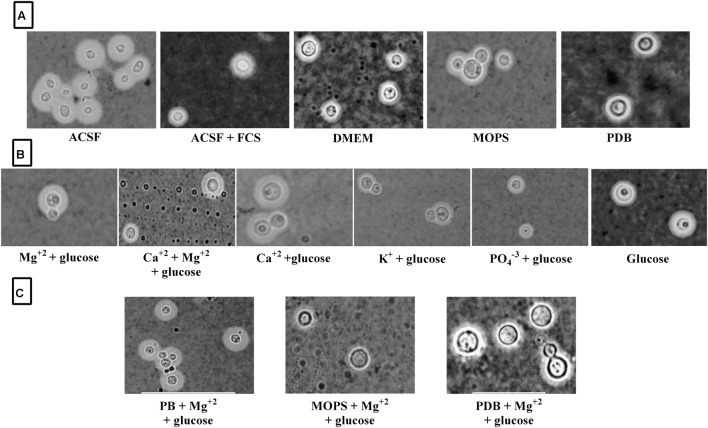
**ATCC *C. neoformans* capsule induction in different media. (A)** Capsule induction in different media; **(B)** Capsule induction in individual ACSF salts; **(C)** Capsule induction in different media supplemented with Mg^2+^ ion.

**FIGURE 8:**
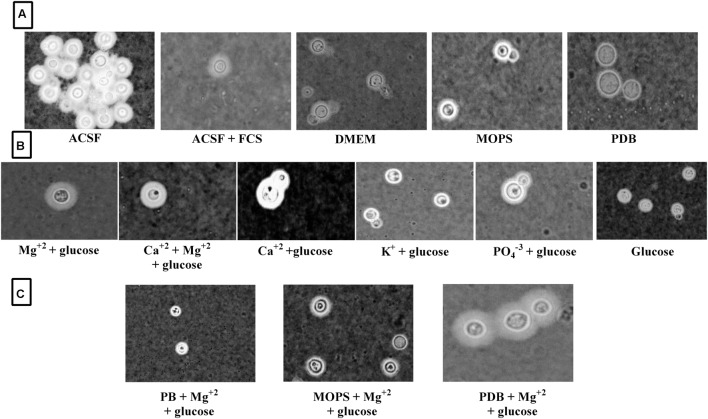
**Clinical *C. neoformans* capsule induction in different media. (A)** Capsule induction in different media; **(B)** Capsule induction in individual ACSF salts; **(C)** Capsule induction in different media supplemented with Mg^2+^ ion.

Since Mg^2+^ in ACSF was shown to increase capsule size, we sought to compare its concentration with human CSF. Mg^2+^ is found at a concentration of 0.97 mM in human CSF and might be a capsule inducing factor that triggers larger capsule size in patients with *cryptococcus* meningitis. Therefore, we also performed Mg^2+^ dose-dependency on *C. neoformans* capsule induction. The media whichever was not having magnesium was selected for the study to confirm that magnesium is one of the important capsule inducing factor. Hence, various concentrations of Mg^2+^ (0–4 mM Mg^2+^) were supplemented in MOPS, PDB, and phosphate buffer. As expected, the medium supplemented with Mg^2+^ produced larger capsule sizes than the control (media without Mg^2+^; **Figure 6**). The dose-dependent studies clearly demonstrate that capsule induction by Mg^2+^ is dose responsive. The optimum concentration required, however, varied between the strains. Dose-dependent analysis of Mg^2+^ on capsule thickness showed that 2.0 mM Mg^2+^ gave the best result for ATCC *C. neoformans*. With increase in Mg^2+^ concentration, there was a small decrease in capsule thickness, and a similar kind of result was observed for clinical *C. neoformans* at 0.5 mM Mg^2+^. This concentration is very similar to the concentration of human CSF (**Figure 6**). *C. neoformans* capsule induction was observed in microscopic analysis for both ATCC and clinical strains, microscopic images showed capsule induction in different media and ions (**Figures 7** and **8**). The One-way ANOVA confirmed that 2.0 mM and 0.5 mM Mg^2+^ concentration is required for significant capsule induction in ATCC *C. neoformans* and clinical *C. neoformans*, respectively (*p* < 0.0001; Supplementary Table 3).

Capsule growth are regulated by multiple pathways and are specifically associated with stress response. cAMP is one such pathway, which is regulated during stress conditions like low glucose, low amino acids, increased CO_2_ (Miwa et al., 2004; Maidan et al., 2005). During stress conditions cAMP levels are elevated and this activates the kinase Pka1, which in turn activates transcriptional factors like Nrg1, Rim101 that are involved in capsule assembly and enlargement, respectively. Mg^2+^ is one of major divalent cation that has important role in cell division, enzyme activation and stress suppression (Nimrichter et al., 2007). It is reported that Mg^2+^ is the most important divalent ion absolutely required for the synthesis of adenylate cyclase which in turn is required for the production of cAMP (Bahn et al., 2004). The cAMP–PKA pathway regulates virulence in *C. neoformans*, including capsule production, melanin formation. From our results we assume that *C. neoformans* when grown in ACSF which has abundant salts and less glucose, activates the cAMP–PKA pathway which are regulated well in the presence of Mg^2+^ ions.

### Biofilm Formation

**FIGURE 9:**
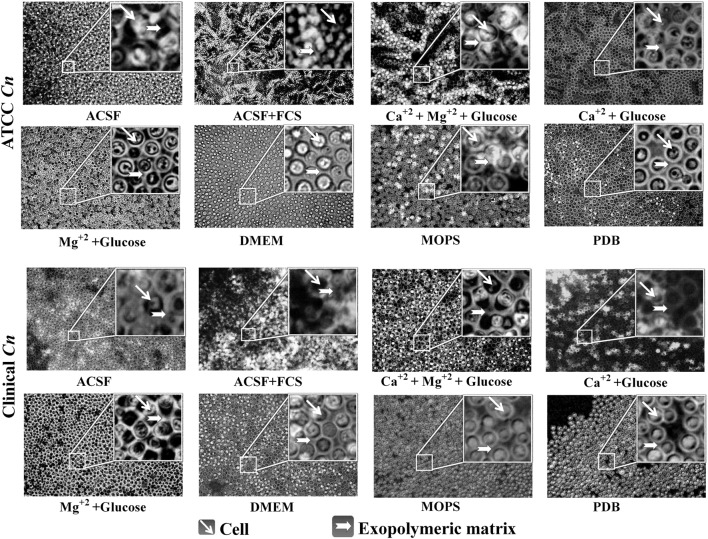
**Biofilm formation on coverslip with different media.** Biofilm observed at 40× light microscope by India ink stain. Images of a mature biofilm show yeast cells and capsular polysaccharide.

*Cryptococcus neoformans* biofilms are becoming common due to the increased use of brain valves and other medical devices. The knowledge on mechanism of biofilm formation by *Cryptococcus* sp. is still in its infancy. The exopolysaccharide of *C. neoformans* renders it to grow as biofilms on various medical devices. We intended to investigate the efficiency of each media to enhance capsule formation which in turn helps *C. neoformans* to grow as biofilm (Martinez and Casadevall, 2007; Robertson et al., 2012). The biofilm formation is shown in **Figure 9**. The result supports our finding that ACSF is the efficient capsule inducing medium for both the strains of *C. neoformans*. Our further studies are intended to form biofilms on different solid supports including medical devices for a better understanding of the role of Mg^2+^ ions in biofilm formation using advanced imaging techniques.

### Capsule Gene Expression Analysis

**FIGURE 10:**
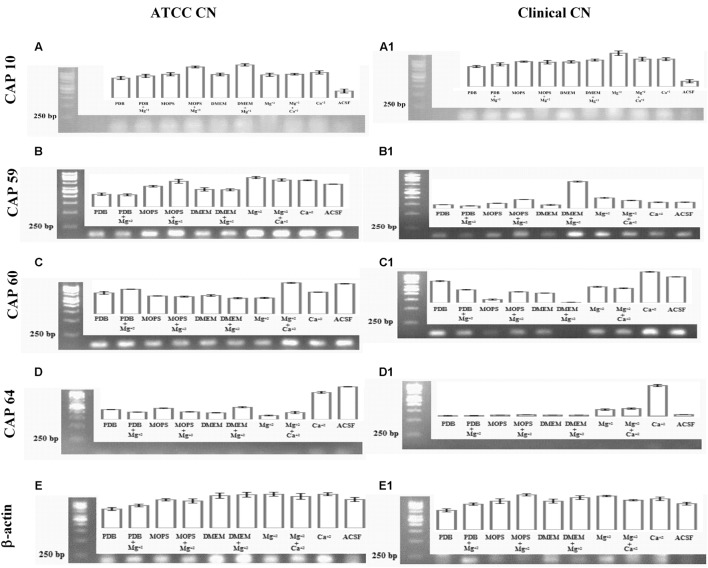
**Capsule associated gene expression.** CAP gene expression of ATCC *C. neoformans*: **(A)** CAP10 gene; **(B)** CAP59 gene; **(C)** CAP60 gene; **(D)** CAP64 gene; **(E)** β-actin gene. CAP gene expression clinical *C. neoformans*: **(A1)** CAP10 gene; **(B1)** CAP59 gene; **(C1)** CAP60 gene; **(D1)** CAP64 gene; **(E1)** β-actin gene.

To look specifically at capsule biosynthesis genes induced by Mg^2+^, we tested the effect of different capsule induction media on the expression of genes important for capsule biosynthesis. We analyzed four major CAP genes namely, CAP10, CAP59, CAP60, and CAP64. Our result (**Figure 10**) on gene expression shows that different media were able to stimulate the expression of CAP10, CAP59, and CAP60, whereas none of the media were able to stimulate the expression of CAP64. These results were similar for both environmental and clinical isolates of *C. neoformans*.

With regard to CAP10, gene, presence of Mg^2+^ in the media lead to enhanced expression of the gene when compared to Mg^2+^-free media, with best expression seen with MOPS + Mg^2+^ in the case of environmental isolate. However, with clinical isolate, presence of Mg^2+^ resulted in lower expression of CAP10 gene when compared to Mg^2+^-free media. Presence of Ca^2+^ did not have any significant effect on CAP10 expression in both isolates. For both environmental and clinical isolates of *C. neoformans*, ACSF did not promote significant CAP10 expression.

For CAP59 expression, again presence of Mg^2+^ in the media leads to increase in expression of the gene in MOPS and DMEM for both environmental and clinical isolates. Presence of Mg^2+^ in PDB did not induce significantly higher CAP59 gene expression in both isolates. Interestingly, CAP59 gene in environmental isolate showed higher expression in ACSF media when compared to clinical isolate.

Unlike CAP59, CAP60 gene expression in clinical isolate was enhanced in the presence of Mg^2+^ in the case of MOPS alone. For this isolate, presence of Mg^2+^ in PDB, and DMEM produced significantly lower levels of CAP60 gene expression. In the case of environmental isolate, presence of Mg^2+^ had a stimulating effect only in the case of PDB, while with MOPS and DMEM, the expression looked almost similar in both Mg^2+^-free and Mg^2+^-containing media. But for both isolates, ACSF induced a strong expression of CAP60 gene.

Interestingly, for both isolates, CAP64 gene expression was not detected in any of the media, including with Mg^2+^, and the reasons for this are unclear. The environmental and clinical isolates displayed differences in expression of capsular polysaccharide under different growth conditions. Interestingly, our study demonstrates that the virulent genes (CAP genes) are highly expressed in the presence of Mg^2+^ ions in both clinical and environmental isolates. Together these results suggest that, Mg^2+^ does have an effect on CAP gene expression, which are important for capsule biosynthesis and virulence.

## Discussion

Meningitis is caused by a variety of microbes, however, those caused by fungus especially *Cryptococcus* is becoming quite common (Karkowska-Kuleta et al., 2009). Cryptococcosis of the CNS is life threatening and presents as meningitis or meningoencephalitis. Initial infection, latency or dissemination of the pathogen is determined by specific morphological features of the microbe. Since infections are rapid in immunocompromised individuals, it strongly suggests the importance of both innate and adaptive immune components (Johnston and May, 2013). Phagocytic cells, especially macrophages of the innate immunity and regulatory arm of the adaptive immune system also appear to be important for cryptococcal clearance (Feldmesser et al., 2001; Taborda and Casadevall, 2002). Capsule is the best-studied virulence factor in *C. neoformans*, wherein it has been shown to inhibit phagocytosis by macrophages, through masking opsonin function (Levitz and DiBenedetto, 1989). Capsule is also important for protecting cryptococci from reactive oxygen species within the lysosome and is also related to proliferative ability of the pathogen inside macrophages (Feldmesser et al., 2001; Zaragoza et al., 2008). Hence studies related to capsule biosynthesis has been a promising topic of interest for *C. neoformans* researchers enabling them to understand capsule synthesis and in turn its pathobiology. Reports have evidently shown that *C. neoformans* cells express antigenic variation in the capsule during murine infection and transmigration through the blood–brain barrier (Jain et al., 2006; Vecchiarelli and Monari, 2012). Thus changes in capsule structure and size are observed during mammalian infection. Since capsule size is highly dynamic based on various environment cues, studies have been carried out under various *in vitro* conditions mimic *in vivo* capsule size changes during infection. Previous studies with mammalian serum, DMEM, MOPS have suggested the capsule production varies with media and growth conditions of *C. neoformans* strains (Haynes et al., 2011; O’Meara and Alspaugh, 2012). Also, a recent study on various nitrogen sources suggested that the capsule growth is induced by urea, which is a prevalent nitrogen source in human CSF (Frazzitta et al., 2013). CSF is widely used as the best diagnostic tool for the identification of *Cryptococcus* sp., as the fungal burden with large capsule size are easily visualized with an India ink stain (Fridlington et al., 2006). A recent interesting study has correlated capsular size in CSF as representative of that found in the brain (Robertson et al., 2014). The predominance of *C. neoformans* in CSF, made us to evaluate the suitability of the CSF as the favorable environment for *C. neoformans* growth. However, we have performed the study for both environmental and clinical isolate. In general, environmental and clinical isolates are different based on their place of origin and habitat. For instance, environmental strains are isolated from soil, pigeon and chicken excreta, trunk hollows of *Tamarindus indica*, and decaying wood. Similarly clinical strains are frequently isolated from CSF of immunocompromised individuals (Emmons, 1951, 1955; Lazéra et al., 1996; Souza et al., 2005). The differences in the genotype, serotype, mating type, and antifungal susceptibility between the environmental and clinical *C. neoformans* has been analyzed by various investigators. Majority of these studies have demonstrated the existence of similarity in genotype, serotype, and mating type in environmental and clinical isolates (Halliday et al., 1999; Campbell et al., 2005; Escandon et al., 2006). However the virulence trait expressions are found to be different between them. For example, phospholipase, urease activity, capsule synthesis, and melanin production are higher in case of clinical isolates, suggesting the significant role of host factors in virulence trait expression. In addition, antifungal susceptibilities are also found to be varied between the clinical and environmental *C. neoformans* (Chowdhary et al., 2011). Thus we studied the responsiveness of environmental and clinical isolates in different growth conditions. The media comprised of various salts like Mg^2+^, Ca^2+^, PO_4_^3-^, K^+^, and with 0.1% of glucose. Our comparative statistical studies with other media like DMEM, MOPS, PDB, and ACSF+FCS showed that capsule induction was significantly higher in ACSF for both the strains. However, one limitation of the present study is the influence of ambient CO_2_ on capsule induction, which could be an effective component under *in vivo* conditions.

The results of capsule induction were also supported by the biofilm formation studies. The higher capsule production enables *C. neoformans* to grow as biofilm and the acapsular mutants are unable to adhere to plastic materials (Martinez and Casadevall, 2006). Though a simple coverslip method was used for the biofilm formation, the biofilm of *C. neoformans* was clearly observed, when grown in ACSF, ACSF salts and other capsule inducing media (**Figure 9**). In a similar kind of study, *C. neoformans* strain B3501 was observed to form a strong biofilm in minimal medium containing glucose (Martinez and Casadevall, 2006).

**FIGURE 11:**
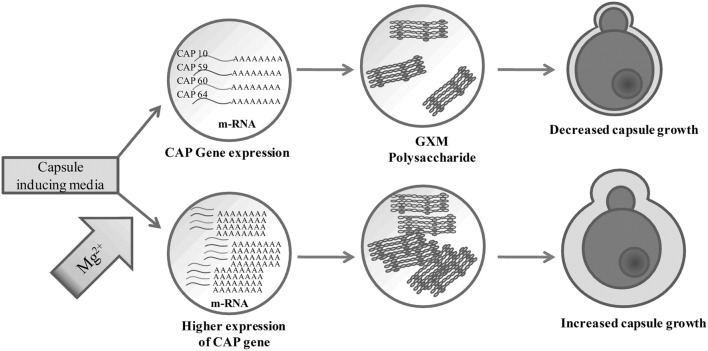
**Proposed role of Mg^2+^ in capsule regulation in *C. neoformans***.

We have extended our study to find the effect of pH and temperature on capsule induction. Though there are reports on the effect of pH and temperature on capsule induction, we also tried similar kind of experiments as the strains selected for the present study are different from those used by other authors. As also suggested by previous reports we could not conclude the role of pH in capsule induction (Gupta and Fries, 2010; O’Meara et al., 2010). The influence of pH varies with the type of media used. Thus to find the significance of pH in capsule induction, expression of capsule biosynthesis genes are required to elucidate the mechanism of capsule induction at different pH. Similarly, the effect of temperature for significant capsule induction varied with the two strains. The capsule production was higher at 37 and 34°C for clinical *Cn* and ATCC *Cn*, respectively. The results show that the temperature required for cell and capsule growth is different for different strains of *C. neoformans*. Zaragoza et al. (2003) reported that temperature does have an effect on cell size but not on capsule size. The authors observed that even a temperature shift from 24 to 37°C, did not affect the proportion of capsule present in the cells. However, from our results, we suggest that temperature does have an effect on capsule growth. Following these results, we used optimized conditions for capsule formation (ACSF, pH 5.5, 72 h, 34°C for ATCC *Cn* and similar conditions at 37°C for clinical *Cn*) to assess the potential of ACSF salts in inducing capsule formation. Similar studies were done previously by some authors to find the significant role of Mg^2+^ + Ca^2+^, PO_4_^3-^, FeSO_4_, and NaCl in capsule formation. PO_4_^3-^ was found to be an essential mineral for nutrient uptake by *C. neoformans* for capsule growth (Kretschmer et al., 2014), and Mg^2+^, and Ca^2+^ ions was found to be important for capsule assembly (Robertson et al., 2012). Whereas the results from our CAP gene expression analyses, suggests the essentiality of Mg^2+^ ion in *C. neoformans* capsule formation. Mg^2+^ ion is one of the most important divalent ion which has many functions including as an enzyme activator. From the previous reports, the significance of cAMP signaling in expression of virulence factors like capsule and melanin are well established. The regulation of cAMP pathway is mediated by adenylate cyclase which are activated in the presence of Mg^2+^ ion (Cramer et al., 2006). Even though Mg^2+^ is identified as the important structural component of biological molecules, the molecular identity of its regulation in yeast cell is poorly elucidated (Udeh et al., 2013). Pathogenicity of *C. neoformans* is in part due to capsule production which is primarily known to block phagocytic response of macrophages (Syme et al., 1999; Del Poeta, 2004; Oliveira et al., 2010). Mutation of four specific genes related to capsule biosynthesis and exocytosis, namely CAP10, CAP59, CAP60, and CAP64 has been shown to results in a acapsular phenotype (Okabayashi et al., 2005; Alarousy et al., 2011; Chun et al., 2011). However, the exact role of these four genes is still unclear, though CAP10 is a glycosyltransferase, CAP59 is important for capsule exocytosis, and CAP60 and CAP64 are probably involved in regulation of capsule biosynthesis (Chang and Kwon-Chung, 1998, 1999; García-Rivera et al., 2004; Chun et al., 2011). The increase in capsule size is associated with increase in virulence and resistance to host immune mediators and thus an understanding of the mechanism of capsule synthesis could be important to identify drug targets for effective therapy. Increase in capsule size has been previously shown to be induced by high CO_2_, low iron and glucose levels, while culture of *C. neoformans* in hypertonic medium containing 1 M NaCl was shown to decrease capsule thickness (Okabayashi et al., 2005; Guimarães et al., 2010). Apart from these reports there are no other studies to the best of our knowledge that have looked at the influence of buffer salts on capsule gene expression. In this study, we specifically looked at the effect of divalent cation Mg^2+^ on capsule gene expression and our results showed that Mg^2+^ had a differential effect on CAP gene expression and this effect was different for both the environmental and clinical isolates of *C. neoformans*. Mg^2+^ had an inducing effect on CAP10 gene of environmental isolate alone and similarly induced the expression of CAP59 in both isolates, while for CAP60, Mg^2+^ was able to induce its expression in clinical isolates. Under certain incubation conditions, Mg^2+^ appeared to have no inducing effect on the CAP genes and this was true in the case of CAP64 gene, wherein we failed to detect its expression in any of the media used, whereas the microscopic analysis revealed increased capsule size, suggesting the involvement of multiple CAP genes in capsule biosynthesis. ACSF on the other hand proved to be effective in inducing CAP59 in environmental isolate. Taken together our result on CAP gene expression (**Figure 10**) shows that the expression of CAP10, CAP59, and CAP60 was enhanced in the presence of Mg^2+^ for both the isolates, suggesting that Mg^2+^ concentration might be critical in regulating capsule synthesis and virulence of *C. neoformans in vivo*, and the exact concentrations of this important divalent cation in blood and CSF could influence the pathogenicity of *C. neoformans* during active infection. However, this remains a critical parameter to be assessed in meningitis patients to correlate with treatment outcome. Our findings on the role of Mg^2+^ ion as an inducing factor for capsule induction (**Figures 11**) could open up new avenues for targeting the capsule biosynthesis pathway in *C. neoformans.*

## Author Contributions

All authors listed, have made substantial, direct and intellectual contribution to the work, and approved it for publication.

## Conflict of Interest Statement

The authors declare that the research was conducted in the absence of any commercial or financial relationships that could be construed as a potential conflict of interest.

## Abbreviations

ACSF: artificial cerebrospinal fluid
*C. neoformans*: *Cryptococcus neoformans*
cAMP: cyclic adenosine monophosphate
CAP gene: capsule associated gene
*Cn*: *Cryptococcus neoformans*
CSF: cerebrospinal fluid
DMEM: Dulbecco’s modified eagle medium
FCS: fetal calf serum
GXM: glucuronoxylomannan
MOPS: 4-morpholinepropanesulfonic acid
PDB: potato dextrose broth
PKA: protein kinase
