# Supplementary material for: Magnesium Ion Acts as a Signal for Capsule Induction in Cryptococcus neoformans
Source: Front Microbiol. 2016 Mar 15;7:325. doi: 10.3389/fmicb.2016.00325 (PMC4791529; doi:10.3389/fmicb.2016.00325)
Supplement: Supplementary file 1 [file Table_1.PDF]

Supplementary Table 1: ANOVA Table for ATCC *Cn* and clinical *Cn* capsule induction in Different Media

**ATCC *Cn***							**Clinical *Cn***				
**Source**	**DF**	**SS**	**MS**	**F**	**P**		**DF**	**SS**	**MS**	**F**	**P**
Media	134	157833.9	1177.9	156.34	0		134	143041.9	1067.5	85.56	0
Error	945	7119.5	7.5				945	11790.7	12.5		
Total	1079	164953.4					1079	154832.6			
S = 2.745			R-Sq = 95.68%	R-Sq(adj) = 95.07%			S = 3.532		R-Sq = 92.38%	R-Sq(adj) = 91.31%	
