# Supplementary material for: Magnesium Ion Acts as a Signal for Capsule Induction in Cryptococcus neoformans
Source: Front Microbiol. 2016 Mar 15;7:325. doi: 10.3389/fmicb.2016.00325 (PMC4791529; doi:10.3389/fmicb.2016.00325)
Supplement: Supplementary file 2 [file Table_2.PDF]

**Supplementary Table 2:** ANOVA Table for ATCC and clinical *Cn* capsule induction in different ACSF salts

| **ATCC *Cn*** | | | | | | | **Clinical *Cn*** | | | | |
|---|---|---|---|---|---|---|---|---|---|---|---|
| **Source** | **DF** | **SS** | **MS** | **F** | **P** | | **DF** | **SS** | **MS** | **F** | **P** |
| Media | 44 | 5765.43 | 131.03 | 33.93 | 0 | | 44 | 5765.43 | 131.03 | 33.93 | 0 |
| Error | 315 | 1216.34 | 3.86 | | | | 315 | 1216.34 | 3.86 | | |
| Total | 359 | 6981.78 | | | | | 359 | 6981.78 | | | |
| | | | | | | | | | | | |
| S = 1.927 | R-Sq = 92.39% | | R-Sq(adj) = 91.32% | | | | S = 1.828 | R-Sq = 94.4% | | R-Sq(adj) = 93.61% | |
