# Supplementary material for: Magnesium Ion Acts as a Signal for Capsule Induction in Cryptococcus neoformans
Source: Front Microbiol. 2016 Mar 15;7:325. doi: 10.3389/fmicb.2016.00325 (PMC4791529; doi:10.3389/fmicb.2016.00325)
Supplement: Supplementary file 3 [file Table_3.PDF]

Supplementary Table 3: ANOVA Table for ATCC and clinical *Cn* capsule induction Different media supplemented with varying Magnesium concentration (0.0 to 4.0mM)

**ATCC *Cn***							**Clinical *Cn***				
**Source**	**DF**	**SS**	**MS**	**F**	**P**		**DF**	**SS**	**MS**	**F**	**P**
Media	26	23920.4	920	77.99	0		26	7759.39	298.44	60.29	0
Error	189	2229.6	11.8				189	935.6	4.95		
Total	215	26150					215	8695			
S = 3.435		R-Sq = 91.47%		R-Sq(adj) = 90.3%			S = 2.225		R-Sq = 89.24%		R-Sq(adj) = 87.76%
